# Year-round tick exposure of dogs and cats in Germany and Austria: results from a tick collection study

**DOI:** 10.1186/s13071-023-05693-5

**Published:** 2023-02-16

**Authors:** Julia Probst, Andrea Springer, Christina Strube

**Affiliations:** Institute for Parasitology, Centre for Infection Medicine, University of Veterinary Medicine Hannover, Buenteweg 17, 30559 Hanover, Germany

**Keywords:** *Ixodes ricinus*, *Ixodes hexagonus*, *Dermacentor reticulatus*, Ticks, Cat, Dog, Pets, Winter, Europe, Geographical distribution

## Abstract

**Background:**

Ticks and tick-borne diseases play a major role in companion animal health. Additionally, the European tick fauna is changing, for instance due to the spread of *Dermacentor reticulatus*, displaying a higher likelihood of winter activity than *Ixodes ricinus*. Therefore, we investigated current tick infestations in dogs and cats in Germany and in parts of Austria and the seasonal infestation risk.

**Methods:**

Overall, 219 veterinary practices were invited to collect ticks from cats and dogs on a monthly basis. Ticks were morphologically identified and female *I. ricinus* specimens were measured to estimate attachment duration.

**Results:**

In total, 19,514 ticks, 17,789 (91.2%) from Germany and 1506 (7.7%) from Austria, were received between March 2020 and October 2021, with 10,287 specimens (52.7%) detached from dogs, 8005 from cats (41.0%) and 1222 from other species (6.3%). In Germany, the most common tick species collected from dogs were *I. ricinus* (78.0%) and *D. reticulatus* (18.8%), while cats mainly harboured *I. ricinus* (91.3%) and *I. hexagonus* (5.5%) and only few *D. reticulatus* (0.6%). In Austria, collected *I. ricinus* reached similar proportions in dogs (90.4%) and cats (95.3%), followed by *D. reticulatus* in both dogs (5.2%) and cats (1.5%), with *I. hexagonus* (0.9%) collected only marginally from cats. The average infestation intensity amounted to 1.62 ticks/dog and 1.88 ticks/cat. The single to multiple infestation ratio was 79.1% to 20.9% in dogs and 69.0% to 31.0% in cats, with cats being significantly more often multiple infested than dogs, while the proportion of mixed-species infestations was 2.0% for both dogs and cats. The average attachment duration of female *I. ricinus* specimens amounted to 78.76 h for dogs and 82.73 h for cats. Furthermore, year-round tick exposure was confirmed, with 108 *D. reticulatus* and 70 *I. ricinus* received on average per month during December 2020 to February 2021.

**Conclusions:**

The study shows a year-round tick infestation risk, with activity of both *D. reticulatus* and *I. ricinus* during winter, and confirms the widespread occurrence of *D. reticulatus* in Germany. Additionally, long average attachment durations and frequent multiple infestations underline the need for adequate year-round tick control, even during the winter months.

**Graphical Abstract:**

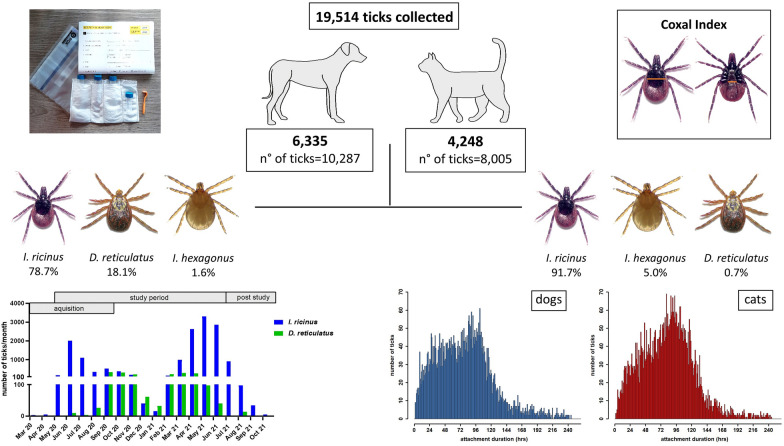

**Supplementary Information:**

The online version contains supplementary material available at 10.1186/s13071-023-05693-5.

## Background

*Ixodes ricinus*, known as the sheep or castor bean tick, and *Dermacentor reticulatus*, known as the meadow or ornate dog tick, are the most common tick species in temperate Europe and act as vectors for various diseases of veterinary importance. Among the most frequent tick-borne diseases (TBDs) of dogs and cats in central Europe is granulocytic anaplasmosis, transmitted by *I. ricinus*. Furthermore, *I. ricinus* acts as a vector for *Borrelia* spirochaetes, causing Lyme borreliosis, and for tick-borne encephalitis virus (TBEV), which may be pathogenic to dogs [1]. Moreover, potentially fatal canine babesiosis transmitted by *D. reticulatus* represents a major threat for dogs [2, 3].

During the last decades, the central European tick fauna has undergone significant changes, with an increasing distribution of ticks across Europe as well as altered activity patterns with a tendency towards year-round activity [4, 5]. While the latitudinal and altitudinal range limit of *I. ricinus* is continuously increasing [6, 7], *D. reticulatus* has undergone a rapid and dramatic range expansion in central Europe, e.g. in Poland [8], the Czech Republic [9] and Germany [5, 10]. The reasons for the geographical spread are not yet fully elucidated and may include global climate change as well as changes in land use and biodiversity [10, 11]. The role of re- and deforestation is currently subject to debate [4, 12], whereas different studies mention an increasing wildlife population as a driving force for the spread of *D. reticulatus* [10, 13]. As *D. reticulatus* prefers open landscapes [14], increasing deforestation and a decrease of agricultural diversity may have contributed to the successful establishment of this tick species in many areas, while increased temperatures facilitate the completion of the tick’s life cycle within 1 year [15]. In addition, more and more dogs are traveling with their owners or are imported from different countries, promoting a spread of tick species across country borders [4, 13]. Related to this expansion of *D. reticulatus*, it is alarming that the incidence of canine babesiosis has increased in Germany and neighbouring countries in recent years [16–18].

In addition to changes in the geographic occurrence of ticks, altered tick activity patterns may result in an increased risk of TBD transmission. Particularly, increasingly mild winters may lead to year-round tick activity [19, 20]. It is commonly assumed that *I. ricinus* starts to be active at soil temperatures above approximately 5–7 °C [21], while *D. reticulatus* is active at a wider temperature range, starting from an air temperature of 0.7 °C and an even lower soil temperature of − 0.2 °C [22], while an increased level of activity has been recorded from 3.8 °C air temperature [23]. In addition, *D. reticulatus* displays high survival rates and activity during winter in the temperate climatic zone [24].

In light of the above-mentioned changes, a nation-wide update regarding tick exposure of dogs and cats is needed to adequately assess the infection risk and improve recommendations for veterinarians and pet owners. Previous studies investigating patterns of tick infestation in dogs and cats in Germany were mostly performed on a local scale and/or date several years back [10, 25, 26]. In the first of these studies, domestic and wild animals from north Baden, in the southwest of Germany, were examined over a 1-year period from 1993 to 1994. Overall, 434 ticks were collected, consisting of 88.7% *I. ricinus* (62.2% from cats and 25.6% from dogs) and 11.3% *Ixodes hexagonus* (3.2% from cats and 1.4% from dogs) [25]. Considering the northern German region of Berlin/Brandenburg, which has been known as *D. reticulatus*-endemic for several years, a study from 2003 indicated that the most frequently collected tick species from dogs was *I. ricinus* (60.8%), followed by a proportion of 11.2% *D. reticulatus* as well as 4.1% *I. hexagonus* [10]. The proportion of *D. reticulatus* on dogs increased to 45.0% in the same area in 2010/2011, while 46.0% of collected ticks were *I. ricinus* and 8.8% *I. hexagonus* [26]. These data indicate that a change in the tick population infesting dogs and cats occurred during the past decades, resulting in an increased or expanded risk of infection with TBDs, respectively. Meanwhile, *D. reticulatus* has been detected all over Germany [5], but the relative frequency on dogs and cats as compared to *I. ricinus* as well as the proportion of other possibly infesting tick species remains unknown on a national level. Therefore, the present study analysed geographic and seasonal patterns of tick exposure of dogs and cats in Germany and Austria based on ticks collected by participating veterinary practices.

## Methods

### Tick collection and morphological identification

Tick collection from infested dogs and cats was designed as convenience sampling by recruiting veterinary practices via sales representatives of Intervet Deutschland GmbH. In each of 27 sales areas, 10 practices were contacted with a focus on an even distribution over rural and urban areas. The contacted veterinary practices received an information folder and a registration form for participation in the study. Recruiting took place 1 month prior to study start and over the course of the first 5 months of the study. Of the contacted practices, a total of 225 agreed to participate, of which 219 participated actively (197 from Germany and 22 from Austria). Tick collection kits containing tick removal tools, 10 tick collection tubes (one tube to be used per animal) and 10 questionnaires each were sent to the participating veterinarians once a month from May 2020 to June 2021, i.e. over a 14-month period. In case > 10 animals were sampled per month, it was possible to reorder kits. For reasons of data protection, it was not possible to record the locations of the participating veterinarians, but the ticks’ geographic origin was obtained via a questionnaire in form of the owner’s postal zip code along with space for further specifications of the area of origin. Questionnaires were matched to the tick collection tubes with a unique identification number. Travel history during the last 2 weeks before tick collection was also documented. In addition, the date of tick detachment was indicated. After every month, collected ticks were sent to the Institute for Parasitology, University of Veterinary Medicine Hannover, where they were morphologically identified under a stereomicroscope (ZEISS Stemi SV 11) according to keys by A.M. Estrada-Peña, D. Andrei and T. Petney [14].

### Determination of tick attachment duration

Female *I. ricinus* specimens were measured using the OLYMPUS cellSens Entry (v. 3.2) software paired with an OLYMPUS SC50 camera adapter to determine the coxal index and estimate the duration of attachment via the formula described by J. Gray, G. Stanek, M. Kundi and E. Kocianova [27]. Values > 245 h were assessed as not reliable and excluded. Due to a lack of data concerning the correlation of morphometric indices with engorgement time for *D. reticulatus*, calculation was not possible for this species and the attachment time was estimated visually. Here, the size increase of the idiosoma was used to classify three stages of engorgement: unengorged (0–24 h), partially engorged (> 24–144 h) and fully engorged (> 144 h), following Fielden et al. [28].

### Tick distribution mapping and statistical analyses

Obtained data were documented in an Excel® spreadsheet (Microsoft Office Professional Plus 16). The geographic distribution of received ticks was plotted using R. v. 4.1.0 [29] with geographic data distributed by OpenStreetMap under the Open Database License (www.openstreetmap.org/copyright).

The distribution of the three most common tick species (*I. ricinus*, *D. reticulatus* and *I. hexagonus*) as well as the proportion of single and multiple infestations was compared between dogs and cats via Chi-square test or Fisher’s exact test if counts in any category were < 5. To examine seasonal differences in the ratio of single and multiple *I. ricinus* infestations between dogs and cats, separate Chi-square tests were carried out for ticks collected from December to February (winter), March to May (spring), June to August (summer) as well as September to November (autumn). *P*-values were corrected for multiple comparisons using the Bonferroni method. Tick attachment duration was compared between dogs and cats via Mann-Whitney *U*-test.

## Results

### Received tick species

In total, 19,514 tick specimens were collected along with respective documentation via questionnaire, 18,126 of them between May 2020 and June 2021, during the originally envisioned 14-month study period. The remaining 1388 ticks were additionally collected from March–April 2020 (10/1,388) and July–October 2021 (1193/1388), or no date of collection was documented on the questionnaire (185/1388).

Most received ticks were identified as *I. ricinus* (15,943/19,514, 81.70%), followed by *D. reticulatus* (2,013/19,514, 10.32%) and *I. hexagonus* (1012/19,514, 5.19%). Furthermore, *Dermacentor marginatus* (38/19,514, 0.19%), *Rhipicephalus sanguineus* (8/19,514, 0.04%), *Haemaphysalis concinna* (9/19,514, 0.05%), *Ixodes canisuga* (7/19,514, 0.04%) and *Ixodes frontalis* (6/19,514, 0.03%) were among the received ticks. The remaining 452 ticks (2.32%) were only identified to genus level because of a deteriorated condition or the difficulty to differentiate between larval and nymphal *I. hexagonus* and *I. canisuga* (precisely 258 *I. hexagonus*/*I. canisuga*, 161 *Ixodes* spp., 43 *Dermacentor* spp. and 4 *Rhipicephalus* spp.).

**Fig. 1:**
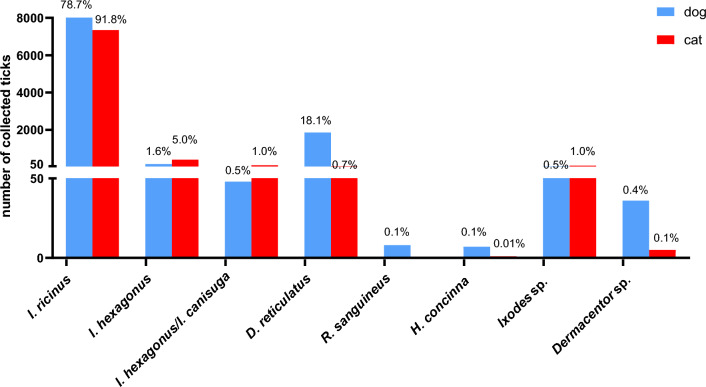
Species distribution of the most common ticks collected from dogs and cats in Germany and Austria. Percentages refer to the proportion for each host species

**Fig. 2:**
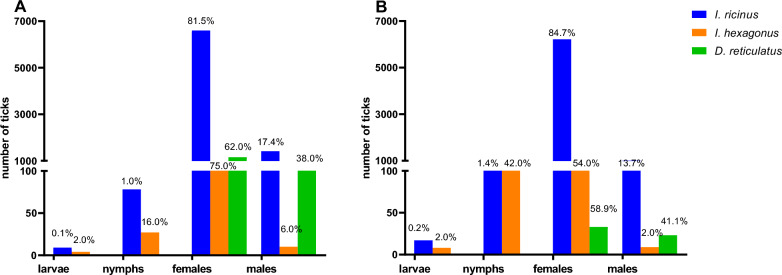
Developmental stage distribution of the three most frequently detected tick species on dogs (**A**) and cats (**B**). Percentages refer to the proportion for each tick species

In terms of host species, 8095 (78.69%) of the ticks found on dogs were identified as *I. ricinus*, 1860 (18.08%) as *D. reticulatus* and 166 (1.61%) as *I. hexagonus* (Fig. 1). Further tick species detected on dogs were *R. sanguineus*, *H. concinna*, *I. canisuga*, *I. frontalis* and *D. marginatus* (Table 1). Of all the ticks found on cats, 7344 (91.74%) were identified as *I. ricinus*, 398 (4.97%) as *I. hexagonus* and 56 (0.70%) as *D. reticulatus* (Fig. 1). This distribution differed significantly compared to dogs (*χ*^2^ = 1555.5, df = 2, *P* < 0.001). Apart from dogs and cats, various other host species contributed a total of 1222 ticks, as summarised in Table 1. Regarding tick developmental stages, only adult *D. reticulatus* specimens were sent in. For *I. ricinus*, 1.07% (87/8095) of specimens found on dogs and 1.67% (123/7344) of specimens from cats were immature stages (larvae or nymphs), respectively (Fig. 2). Regarding *I. hexagonus*, 18.67% (31/166) of the specimens detached from dogs and 43.72% (174/398) of those from cats were immature (Fig. 2). Immature stages of *I. ricinus* (larvae: 30/15,913; 1.89%; nymphs: 347/15,913; 2.18%) and *I. hexagonus* (larvae: 12/1012; 1.19%; nymphs: 226/1012; 22.33%) were mainly sent in from Germany, while only 2 larvae and 10 nymphs of *I. ricinus* were received from Austria.

**Table 1:** Overview of tick specimens collected from the various host species

	*I. ricinus*	*I. hexagonus*	*I. canisuga*	*I. hexagonus / I. canisuga*	*I. frontalis*	*D. reticulatus*	*D. marginatus*	*R. sanguineus*	*H. concinna*	*Ixodes* spp.	*Dermacentor* spp.	*Rhipicephalus* spp.	Total no.
Dog	8095	166	4	48	1	1860	1	8	7	56	36	2	10,284
Cat	7344	398	2	107	0	56	0	0	1	83	5	1	7997
Hedgehog	107	438	0	102	0	0	0	0	0	9	0	0	656
Horse	39	1	0	0	0	76	30	0	0	1	2	0	149
Rabbit	121	0	0	0	0	0	0	0	0	9	0	0	130
Deer	27	0	0	0	0	0	0	0	1	0	0	0	28
Human	15	1	0	0	0	13	1	0	0	1	0	0	31
Sheep	17	0	0	0	0	0	5	0	0	0	0	0	22
Cattle	13	0	0	0	0	0	1	0	0	0	0	0	14
Bird	6	0	0	0	5	0	0	0	0	1	0	0	12
Other*	28	1	1	0	0	0	0	0	0	0	0	0	30
Host unknown	131	7	0	1	0	8	0	0	0	1	0	1	149
Total	15,943	1012	7	258	6	2013	38	8	9	161	43	4	19,502

^*^Other host species: *I. ricinus*: badger (*n* = 4), guinea pig (*n* = 8), hamster (*n* = 3), squirrel (*n* = 3), fox (*n* = 2), hare (*n* = 2), rat (*n* = 1), goat (*n* = 1), marten (*n* = 4); *I. hexagonus*: marten (*n* = 1); *I. canisuga*: badger (*n* = 1)

Twelve ticks are not listed as their deteriorated condition did not allow morphological identification

### Geographical distribution of received ticks

**Fig. 3:**
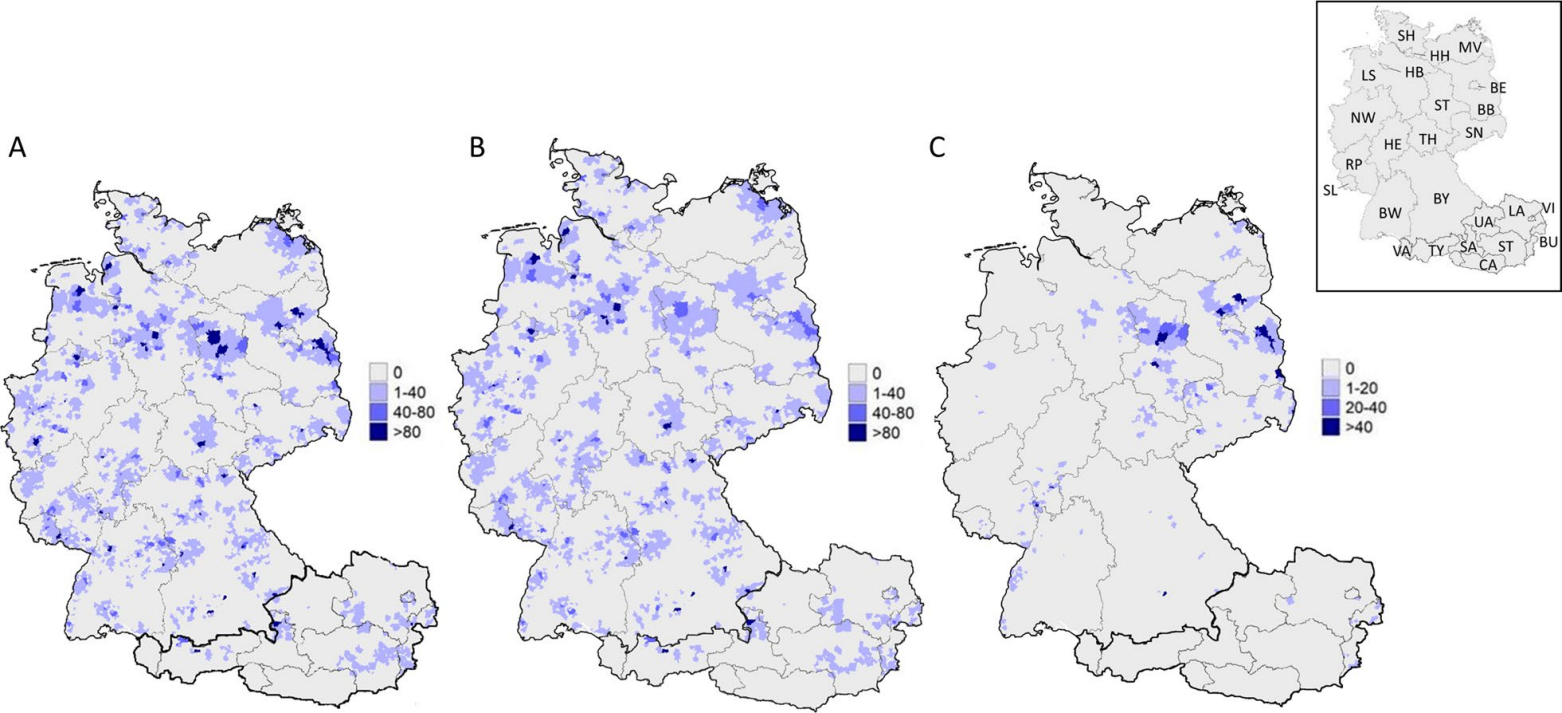
Geographical distribution (zip code areas) and number of all ticks (**A**), *I. ricinus* (**B**) and *D. reticulatus* (**C**) detached from dogs and cats in Germany and Austria. A map of the federal states of Germany and Austria is shown in the upper right corner (Germany: BW = Baden-Württemberg, BY = Bavaria, BE = Berlin, BB = Brandenburg, HB = Bremen, HH = Hamburg, HE = Hesse, LS = Lower Saxony, MV = Mecklenburg-Vorpommern, NW = North Rhine-Westphalia, RP = Rhineland-Palatinate, SL = Saarland, SN = Saxony, ST = Saxony-Anhalt, SH = Schleswig–Holstein, TH = Thuringia; Austria: BU = Burgenland, CA = Carinthia, LA = Lower Austria; UA = Upper Austria, SA = Salzburg; ST = Styria, TY = Tyrol, VA = Vorarlberg, VI = Vienna)

Most of the collected ticks were sent in from Germany (17,789/19,514; 91.16%) and Austria (1,506/19,514; 7.72%). In addition, some sporadic submissions from France (15/19,514; 0.08%), The Netherlands (3/19,514; 0.02%) and Switzerland (3/19,514; 0.02%) were received, while the country and postal code of 198/19,514 submissions (1.01%) was not indicated. The distribution of *I. ricinus* and *D. reticulatus* per postal code area is shown in Fig. 3. As expected, *I. ricinus* was received from all participating veterinarians. Due to the location of recruited practices, some clustering (> 50 ticks per postal code area) in the German federal states of Lower Saxony and Bavaria was observed. Regarding the federal state of Mecklenburg-Western Pomerania, only few ticks were received because only a few veterinarians from the northeast of this federal state participated in the study.

**Fig. 4:**
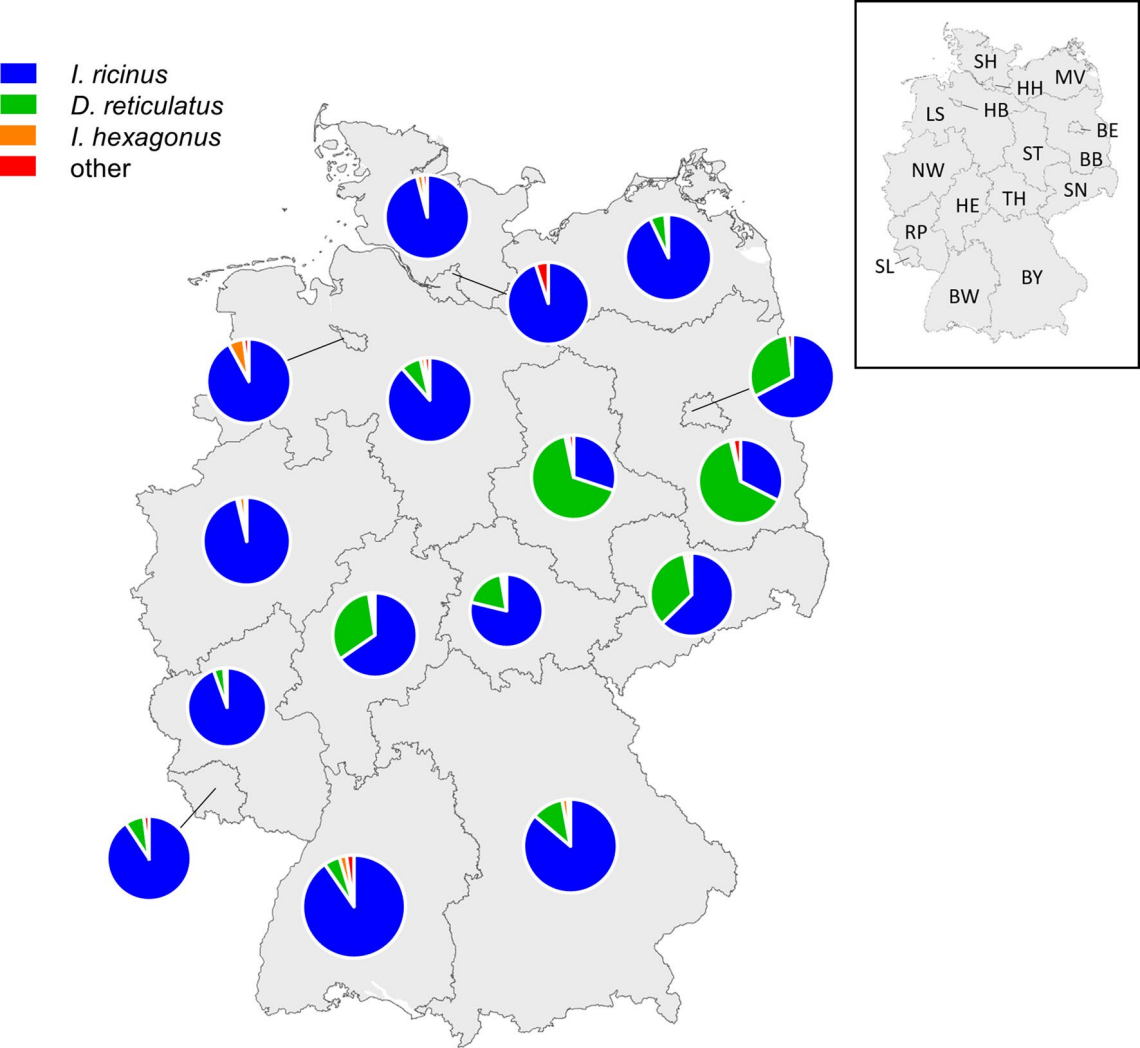
Proportions of the three most frequent tick species detached from dogs per German federal state. A map of the federal states of Germany is shown in the upper right corner (Germany: BW = Baden-Württemberg, BY = Bavaria, BE = Berlin, BB = Brandenburg, HB = Bremen, HH = Hamburg, HE = Hesse, LS = Lower Saxony, MV = Mecklenburg-Vorpommern, NW = North Rhine-Westphalia, RP = Rhineland-Palatinate, SL = Saarland, SN = Saxony, ST = Saxony-Anhalt, SH = Schleswig-Holstein, TH = Thuringia)

Except for Schleswig-Holstein and the two city states Hamburg and Bremen in the northernmost part of Germany, from where fewer ticks were received overall because of few participating veterinarians, *D. reticulatus* was also sent in from all German federal states. Primarily, *D. reticulatus* specimens were sent in from the east of Germany, where the proportion of *D. reticulatus* among ticks found on dogs reached up to 66.67% (federal state of Saxony-Anhalt, Table 2, Fig. 4). Among the remaining federal states, this proportion varied between 0.99 and 63.59%. Regarding cats, the proportion of *D. reticulatus* varied between 0 and a maximum of 1.81% (federal state of Saxony-Anhalt, Table 2). The infestation rates with the most frequently collected tick species among tick-infested dogs and cats in the different German and Austrian federal states are given in Additional file 1: Table S1.

**Table 2:** Overview of the distribution of the most frequently collected tick species from cats and dogs over the German federal states (number per tick species/% of total ticks)

	Dogs				Cats			
	*I. ricinus*	*D. reticulatus*	*I. hexagonus*	Total	*I. ricinus*	*D. reticulatus*	*I. hexagonus*	Total
Baden-Württemberg	763/90.40%	42/4.98%	19/2.25%	824/97.63%	527/85.28%	1/0.16%	61/9.87%	589/95.31%
Bavaria	1,190/86.17%	151/10.93%	27/1.96%	1,368/99.06%	1,370/87.48%	1/0.06%	145/9.26%	1,516/96.81%
Berlin	70/67.31%	32/30.77%	0/0.00%	102/98.08%	61/96.83%	1/1.59%	0/0.00%	62/98.41%
Brandenburg	282/32.49%	552/63.59%	9/1.04%	843/97.12%	388/94.87%	0/0.00%	10/2.44%	398/97.31%
Bremen	93/92.08%	0/0.00%	6/5.94%	99/98.02%	30/90.91%	0/0.00%	3/9.09%	33/100%
Hamburg	35/94.59%	0/0.00%	0/0.00%	35/94.59%	24/100%	0/0.00%	0/0.00%	24/100%
Hesse	454/65.42%	223/32.13%	9/1.30%	686/98.85%	317/96.35%	2/0.61%	7/2.13%	326/99.09%
Lower Saxony	1,188/88.59%	104/7.76%	23/1.72%	1,315/98.06%	821/94.69%	0/0.00%	28/3.23%	849/97.92%
Mecklenburg-Western Pomerania	210/92.92%	13/5.75%	2/0.88%	225/99.56%	184/85.19%	0/0.00%	29/13.43%	213/98.61%
North Rhine-Westphalia	1,265/96.27%	13/0.99%	27/2.05%	1,305/99.32%	693/95.98%	1/0.14%	15/2.08%	709/98.20%
Rhineland-Palatinate	560/94.44%	25/4.22%	4/0.67%	589/99.33%	502/94.36%	2/0.38%	9/1.69%	513/96.43%
Saarland	136/90.67%	11/7.33%	0/0.00%	147/98.00%	113/89.68%	0/0.00%	11/8.73%	124/98.41%
Saxony	338/62.71%	185/34.32%	8/1.48%	531/98.52%	576/94.89%	2/0.33%	13/2.14%	591/97.36%
Saxony-Anhalt	166/30.07%	368/66.67%	8/1.45%	542/98.19%	253/91.34%	5/1.81%	18/6.50%	276/99.64%
Schleswig-Holstein	119/95.95%	0/0.00%	9/2.28%	388/98.23%	309/97.17%	0/0.00%	2/0.63%	311/97.80%
Thuringia	379/78.81%	28/18.54%	2/1.00%	149/98.68%	133/75.14%	0/0.00%	29/16.38%	162/91.53%
Total*	7,248/78.02%	1,747/18.81%	153/1.65%	9,148/98.47%	3,601/91.53%	15/0.22%	380/5.52%	6,696/97.27%

^*^Percentages refer to the whole of Germany

Due to the lower number and uneven distribution of recruited veterinary practices in Austria, ticks were mostly sent in from five federal states, namely Burgenland, Salzburg, Styria, Lower Austria and Tyrol, with sporadic collections in other areas. Regarding *D. reticulatus*, 91.15% (43/47) of the Austrian specimens were received from the state of Burgenland, with 22 specimens (46.81%) found in only one postal code area (7023). Most *I. ricinus* submissions came from Salzburg (284/1,375; 20.65%), Styria (271/1,375; 19.71%), Burgenland (261/1,375; 18.98%) and Lower Austria (223/1,375; 16.22%), so that other parts of Austria may be underrepresented, since only a few veterinarians participated in Austria. An overview of the distribution of the most frequently collected tick species from cats and dogs over the Austrian federal states is given in Additional file 2: Table S2.

### Characterisation of tick origin

Only a few owners gave specific information on animal husbandry, namely that the animal only had access to their yard or garden (280/18,295; 1.53%) or lives near the coast (43/18,295; 0.23%).

Most of the ticks were probably acquired in the declared postal code area, as the host animals had not left this region during the last 2 weeks before tick collection (16,178/19,514; 82.90%). While 6.90% (1,347/19,514) of host animals had a travel history beyond the given postal code area or even beyond the German border, in 10.19% (1,989/19,514) of cases, no data on travel history were available.

### Infestation intensity and co-infestations in dogs vs. cats

In total, 6335 dogs were part of this submission study and provided a total of 10,287/19,514 ticks, so that the average infestation was 1.62 ticks per dog. Of all dogs, 5011 (79.10%) were infested with a single tick only and 1324 (20.90%) with more than one tick, whereby the maximum number of detached ticks from one dog amounted to 96. This dog was reported as a Labrador-Husky mix co-infested with 91 adult *I. ricinus* and 5 adult *D. reticulatus*.

Concerning cats, 4248 animals were sampled and provided a sample size of 8,005/19,514 ticks, so that the average infestation was 1.88 ticks per cat. The number of single infestations amounted to 2932 (69.02%), while 1316 cats (30.98%) were infested with multiple ticks. The proportion of multiple infestations on cats was significantly higher compared to dogs (*χ*^2^ = 137.45, df = 1, *P* < 0.001). The highest infestation intensity was recorded on a European shorthair, which was infested with 54 *I. hexagonus* specimens, including 50 nymphs and four females.

**Fig. 5:**
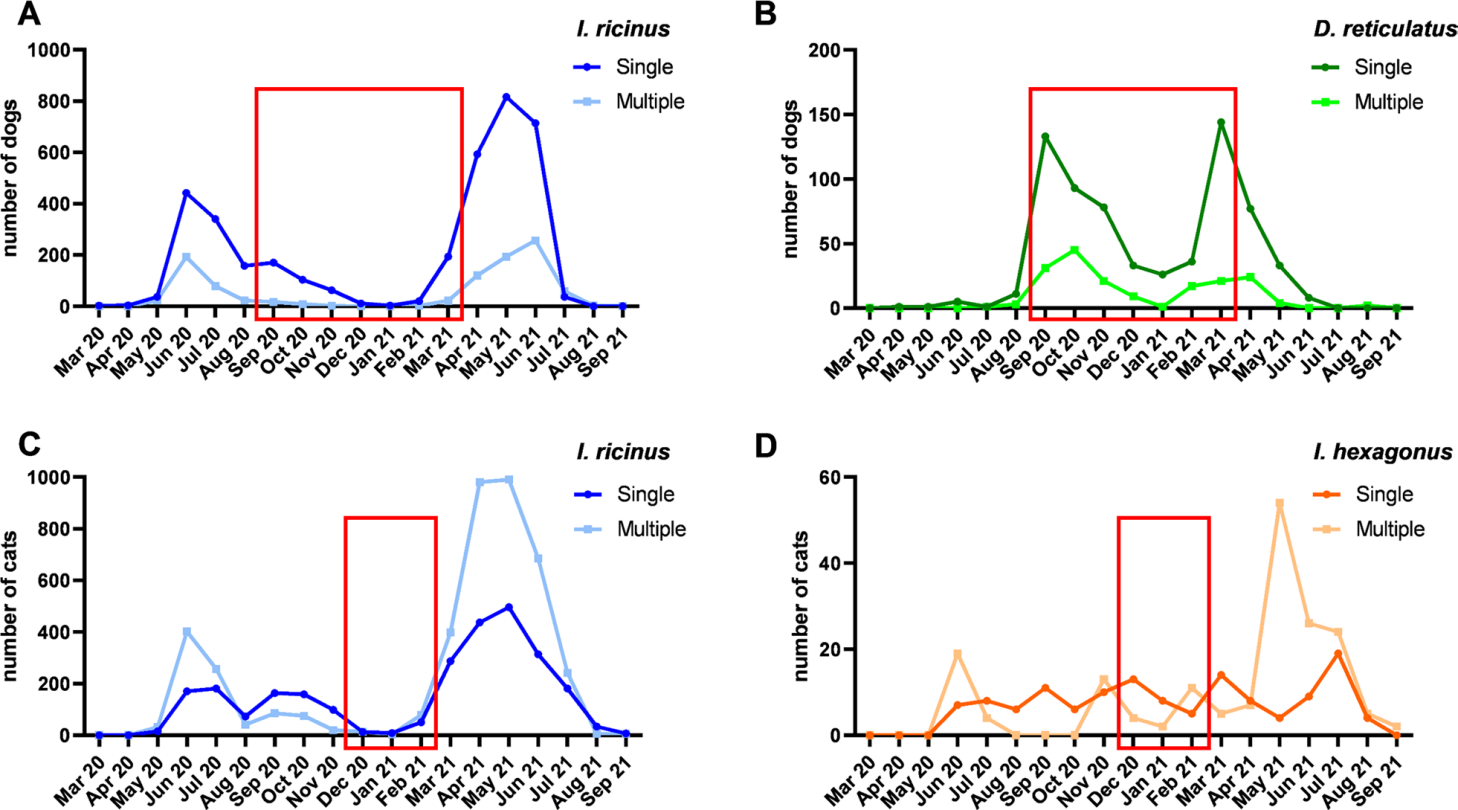
Multiple and single infestations with the two most frequently collected tick species on dogs (*I. ricinus* [**A**] and *D. reticulatus* [**B**]) and cats (*I. ricinus* [**C**] and *I. hexagonus* [**D**]) over the course of the study. Red boxes illustrate the main periods of tick species-specific differences

The seasonal pattern of infestations with multiple vs. single specimens of the most frequently detected tick species (i.e. *I. ricinus* and *D. reticulatus* for dogs and *I. ricinus* and *I. hexagonus* for cats) is visualised in Fig. 5. Regarding *I. ricinus*, both single and multiple infestations peaked during the period of main tick activity, which was from May to July (Fig. 5A, C). Concerning *D. reticulatus*, also both infestation types peaked during the tick’s main activity periods, e.g. September to October 2020 as well as in March to April 2021 (Fig. 5B). In contrast, single infestations with *I. hexagonus* in cats occurred throughout the study period without distinct peaks, while infestations with multiple specimens peaked in May 2021 (Fig. 5D). While in dogs the number of single infestations was always above the level of multiple infestations, cats were infested with multiple ticks more often in the times of species-related activity peaks (Fig. 5C, D). Cats had a significantly higher proportion of multiple infestations with *I. ricinus* than dogs in each season of the year (Table 3).

**Table 3:** Comparison of single and multiple *I. ricinus* infestations of cats and dogs over the study period (spring = March–May; summer = June–August; autumn = September–November; winter = December-February), with Bonferroni-corrected *P*-values

	Dogs		Cats		χ^2^	df	*P*-value	Performed test
	Single infestation	Multiple infestation	Single infestation	Multiple infestation				
Spring 2020	44/65.67%	23/34.33%	15/31.91%	32/68.09%	11.291	1	< 0.001	Chi-square test
Summer 2020	940/76.11%	295/23.89%	424/37.72%	700/62.28%	354.04	1	< 0.001	Chi-square test
Autumn 2020	337/92.33%	28/7.67%	422/70.10%	180/29.90%	65.195	1	< 0.001	Chi-square test
Winter 2020/2021	34/94.44%	2/5.56%	72/42.60%	97/57.40%	na	na	< 0.001	Fisher’s exact test
Spring 2021	1,603/82.67%	336/17.33%	1,220/34.00%	2,368/66.00%	1191.2	1	< 0.001	Chi-square test
Summer 2021	714/69.12%	319/30.88%	529/36.18%	933/63.82%	261.33	1	< 0.001	Chi-square test
Autumn 2021*	23/95.83%	1/4.17%	9/64.29%	5/35.71%	na	na	0.01849	Fisher’s exact test

^*^Contains only data of September and October 2021. *na* not applicable

The seasonal comparison of single and multiple infestations between the two most frequently collected tick species per host showed significant differences in dogs. The proportion of multiple infestations by *D. reticulatus* was significantly higher compared to *I. ricinus* in the autumn of 2020 (*χ*^2^ = 47.092, df = 1, *P* < 0.001), winter of 2020/21 (χ^2^ = 21.886, df = 1, *P* < 0.001) and spring 2021 (Fisher’s exact test, *P* < 0.001), while in summer 2021 the opposite pattern occurred (*χ*^2^ = 9.3044, df = 1, *P* < 0.001). For cats, no significant difference between seasonal rates of multiple infestations with *I. ricinus* and *I. hexagonus* was recorded (*P* > 0.05).

**Fig. 6:**
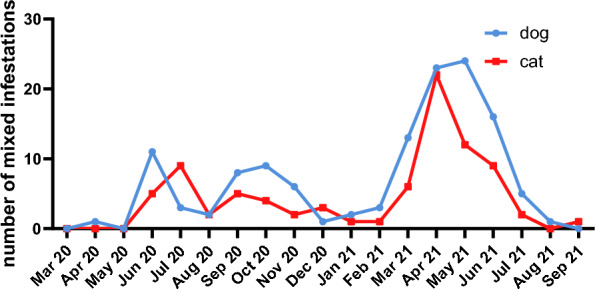
Number of infestations with more than one tick species on dogs and cats per study month

Most multiple infestations were limited to one tick species, while fewer mixed-species infestations were also observed, namely in 128 (2.02%) dogs and 84 (1.98%) cats. These showed a similar pattern in dogs and cats throughout the study and peaked between March and June 2021 (Fig. 6). Regarding the different tick species in these mixed infestations, in dogs almost as many *D. reticulatus* (40.1%) as *I. ricinus* (48.5%) specimens were found, followed by some *I. hexagonus* (3.2%). In cats, the dominating species was *I. ricinus* (59.3%), followed by *I. hexagonus* (17.1%) and not further determinable *Ixodes* sp. (9.9%).

### Attachment duration

**Fig. 7:**
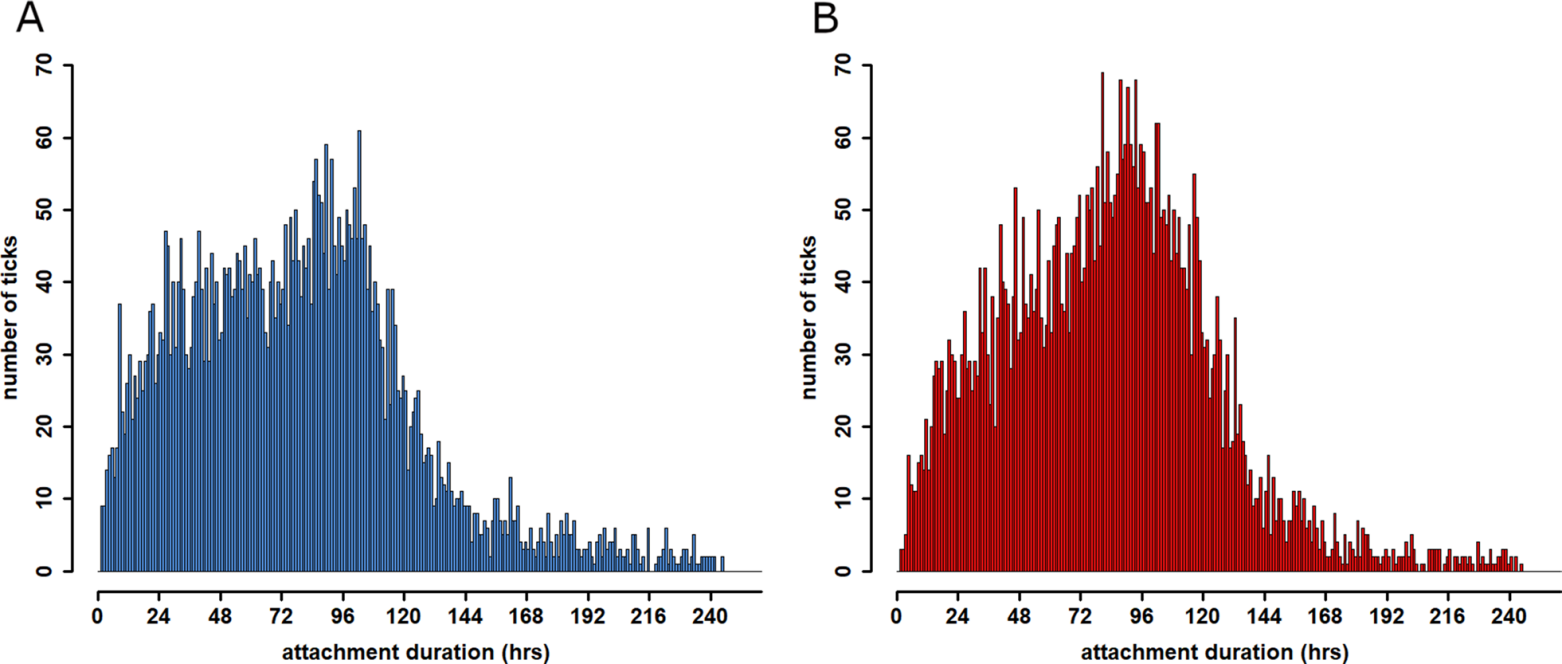
Distribution of attachment time of female *I. ricinus* specimens collected from dogs (**A**) and cats (**B**)

Attachment duration was calculated for 10,871 female *I. ricinus*, 5175 of which were collected from dogs and 5544 from cats. For another 1421 specimens collected from dogs (21.54%) and 674 of those from cats (10.84%), the attachment time was not determinable because of calculation limits resulting in extreme values; therefore, these ticks were excluded. Dogs infested with female *I. ricinus* specimens harboured these for 78.76 h on average (standard deviation [SD] = 45.22 h, median = 76.85 h). In cats, the average attachment time was slightly higher (Mann-Whitney U-test, χ^2^ = 13,300,506*, P* < 0.001), with an average of 82.73 h (SD = 41.65 h, median = 82.94 h; Fig. 7).

Among the 1159 female *D. reticulatus* specimens collected from dogs, 42.02% (487/1159) were fully engorged, 21.92% (254/1159) were partially engorged and 35.98% (417/1159) were detached before engorgement started, while for one specimen the stage of engorgement was impossible to determine because of the tick’s condition. In cats, the distribution was similar although the sample size was considerably lower. Of the 33 collected females, 45.45% (15/33) were fully engorged, 21.21% (7/33) were partially engorged and 33.33% (11/33) were unengorged.

### Temporal course of *I. ricinus* and *D. reticulatus* collection

**Fig. 8:**
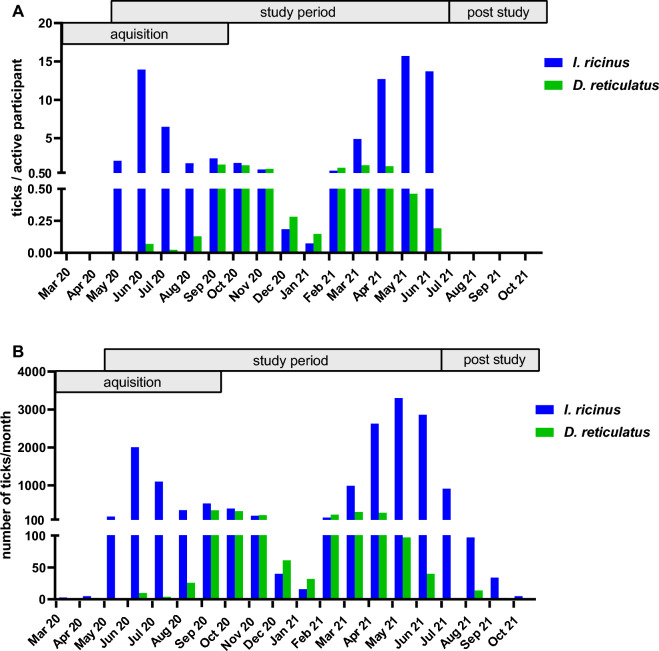
Collected *I. ricinus* and *D. reticulatus* specimens per actively participating veterinarian over the course of the study period (**A**) and total number of received ticks per month (**B**). The number of specimens per active participant is not indicated for March to April 2020 or July to October 2021, as these periods were outside the 14-month study period and no collection kits were sent out for these months

To account for the fact that the number of actively participating veterinary practices varied between individual months, the monthly number of received ticks was divided by the number of active participants in that month (Fig. 8a). The study started in May 2020 with 86 participating veterinarians and resulted in a maximum number of 219 recruited participants in September 2020, 197 from Germany and 22 from Austria. After September 2020, no new participants were recruited and the number of actively participating veterinarians, meaning all participants that did not sign off for the month and got a new collection kit, never dropped below 200 during the predetermined study period up to June 2021 (Fig. 8). In July 2021, just 160 veterinarians were supplied with tick collection boxes because only remaining kits were sent out. However, veterinarians may have used remaining boxes from the preceding months, as evidenced by the fact that some ticks were still sent in after the previously determined study period from July to October 2021 (1193/19,514; 6.11%). Therefore, the number of ticks per actively participating veterinarian was not calculated as of July 2021. Concerning the main study period, it cannot be excluded that some veterinarians were not taking part during some months despite having ordered boxes. Nevertheless, normalising tick numbers by the recorded number of participants per month was regarded to yield a more accurate estimate of tick activity than normalising by incoming boxes because empty boxes may not have been sent to our institute.

Both *I. ricinus* and *D. reticulatus* were collected throughout the year (Fig. 8). Regarding the temporal distribution of received *I. ricinus* ticks, two major peaks were evident, namely in June 2020 (2007 ticks received in total, 13.93 per participant) and May 2021 (3300 ticks received in total, 15.71 per participant). A smaller peak was also observed in September 2020 (524 ticks received in total, 2.00 per participant). Over the winter period from December 2020 to February 2021, an average number of 70 ticks per month was sent in (0.34 ticks per participant).

Regarding *D. reticulatus*, collections peaked in September 2020 (345 ticks received in total, 1.56 per participant) and in March 2021 (300 ticks received in total, 1.49 per participant). Over the winter period (December 2020–February 2021), an average of 108 *D. reticulatus* specimens was sent in per month (0.53 ticks per participant).

## Discussion

The present study constitutes the first large-scale investigation of tick infestation of cats and dogs from all over Germany and parts of Austria. Previous studies have investigated patterns of tick infestation on a local scale and mainly concentrated on dogs, e.g. in southwestern Germany [25] or in the Berlin/Brandenburg area, focusing on the regional importance of *D. reticulatus* [10, 26]. In the light of climate change and altered tick activity patterns/species distributions, a large-scale study on the year-round tick infestation risk was urgently needed. Therefore, the present study also included the winter period. The fact that almost 20,000 ticks were obtained during the 14-month study period indicates that many dogs and cats are inadequately protected. Therefore, recommendations for tick control as well as counselling of animal owners need to be improved to protect cats and dogs from infection with TBDs as well as to slow the spread and increasing local abundance of *D. reticulatus* on a national and European scale.

### Frequently collected tick species and their geographic distribution

As expected, most ticks collected from dogs and cats (81.7%) were identified as *I. ricinus*. However, *D. reticulatus* represented the second most common tick species, accounting for one in 10 ticks (10.3%) in total and for almost every fifth tick (18.1%) collected from dogs. This is in line with the fact that this tick species is now widely spread over Germany, with some highly endemic areas in eastern Germany [5]. Accordingly, *D. reticulatus* was received from all federal states, except for the northernmost part of the country (federal state of Schleswig-Holstein, city states of Bremen and Hamburg). These northernmost areas together comprise only approximately 6.5% of the German population [30] and were therefore sampled less intensely, with only 273 ticks collected from dogs compared to other northern federal states like Lower Saxony, represented by 1384 ticks collected from dogs. Therefore, further local investigations are necessary to assess the abundance and distribution of tick species in these most northern areas. Most *D. reticulatus* specimens originated from the eastern federal states (Saxony-Anhalt, Saxony, Brandenburg and Berlin) and the Rhine Valley, which represent the original distribution areas of *D. reticulatus* in Germany [31]. In 2013, a study in Brandenburg already determined a proportion of 45.0% *D. reticulatus* specimens among all ticks detached from dogs, which almost corresponded to the percentage of *I. ricinus* (46.0%) [26]. In the present study, the proportion of *D. reticulatus* on dogs amounted to 55.0% in Brandenburg and ranged from 1.0% in the federal state of North Rhine-Westphalia to 66.0% in Saxony-Anhalt. In the highly *D. reticulatus*-endemic regions, this tick species thus seems to displace *I. ricinus* as the most common tick species parasitising dogs, which is alarming in light of the vector function for *Babesia canis*. An increasing canine babesiosis incidence has already been observed in the Berlin/Brandenburg area [32] and in the federal state of Hesse [18], underlining the need for proper tick control. In this context, differences in the duration of action of acaricides against different tick species need to be considered. For example, several permethrin-based products have a 4-week duration of action against *I. ricinus* but only 3 weeks against *D. reticulatus*, so more frequent treatment in *D. reticulatus*-endemic areas is necessary. Some fipronil- or deltamethrin-based products are not licensed against *D. reticulatus* at all. A thorough counselling of pet owners by veterinarians concerning the different acaricides is therefore necessary to achieve adequate protection.

Regarding Austria, only 22 veterinary practices participated in the current study, so it was not possible to assess a nation-wide geographic distribution pattern for Austria. Nevertheless, only 3.1% of all ticks from Austria were identified as *D. reticulatus* compared to every 10th tick from Germany (10.9%). These numbers are comparable with German areas characterised by low *D. reticulatus* abundance as well as with numbers from Switzerland [33]. Most of the Austrian *D. reticulatus* specimens originated from the eastern part of the country, which is already known as a *D. reticulatus*-endemic area [34, 35].

The comparably low proportion of *D. reticulatus* obtained from cats (0.70%) reflects the affinity of *D. reticulatus* for canine hosts. Similarly, only 0.6% of 2535 respectively 0.4% of 1960 *D. reticulatus* specimens from two citizen science studies were detached from cats compared to 47.8% respectively 66.9% from dogs [5, 36]. Consequently, *I. hexagonus*, with a share of 5.0%, was the second most common tick species after *I. ricinus* (91.7%) found on cats and the third most common species found on dogs (1.6%). Previous studies also identified these three tick species as the most frequently detached ticks from dogs in Germany [10, 26] and further central European countries [33, 37–39], while cats have only rarely been studied in this regard [25, 39].

In cats, the comparably high *I. hexagonus* proportion in relation to dogs is probably driven by its species-specific behaviour [40]. Due to their nocturnal activity, often unrestricted hunting drive and smaller body size, cats probably come into contact with hedgehogs and their nests more often than dogs. Interestingly, in a study by Dautel et al. [10], 4.1% of ticks detached from dogs were identified as *I. hexagonus*, while this proportion amounted to only 1.6% in the current study. This may be due to the increased proportion of *D. reticulatus* on dogs in the present study, which amounted to only 9.1% in the study by Dautel et al. [10].

Related to the differences in tick species distribution, mixed-species infestations were approximately detected as often in dogs (2.02%) as in cats (1.98%). For both cats and dogs, the distribution of mixed-species infestations throughout the year followed the activity peak of *I. ricinus*. In dogs, an additional increase during the main activity period of *D. reticulatus* in October and November was observed.

### Further tick species

Apart from *I. ricinus, D. reticulatus* and *I. hexagonus*, several other Ixodidae were sporadically sent in. These included ticks primarily associated with wildlife, such as *I. frontalis*, *I. canisuga* and *H. concinna*, which were rarely detected on dogs and cats. Of note, three of seven received *H. concinna* specimens were detached from hunting dogs. Furthermore, *D. marginatus* was received primarily from horses, humans, sheep and cattle. The predominant association with hoofed animals was not surprising as these are known as preferred hosts for adult *D. marginatus* [5, 9, 14]. Similarly, 35.0% of *D. marginatus* received in the frame of a citizen science study were detached from horses but only 3.04% from dogs [5].

Moreover, *R. sanguineus*, known as the brown dog tick, plays a role as an imported tick species in Germany and Austria [41]. As expected, *R. sanguineus* was found exclusively on dogs, mainly on those with a travel history to Bosnia, Bulgaria and Hungary, where this tick species is widely distributed [14, 42]. Although only few *R. sanguineus* specimens were received, these findings show that import by travelling dogs occurs on a regular basis. In central Europe, *R. sanguineus* may reproduce inside buildings, which should be prevented by adequate tick prophylaxis and monitoring of travelling dogs or dogs imported by animal welfare organisations or private individuals, respectively.

### Tick developmental stages

Regarding the tick developmental stages, the results are similar to previous tick submission studies from Germany and other European countries with the same collected tick species [26, 38]. While for *I. ricinus* only 2.3% of all received specimens were nymphs or larvae, this proportion amounted to 23.5% for *I. hexagonus* collected from dogs and even to 43.7% of those collected from cats. Similarly, foxes also harbour a much larger proportion of *I. ricinus* adults than nymphs [43], in contrast to *I. hexagonus* and *I. canisuga* [44], indicating that host preference may play a role regarding this pattern. In addition, single infestations with immature stages of *I. ricinus* may be harder to detect because of the ticks’ small size, while infestations with immature stages of nidicolous ticks are more likely to result in higher infestation intensities. Such high-intensity infestations are less likely to be missed; as a result, the proportion of *I. ricinus* immature stages present on dogs and cats may be underestimated compared to *I. hexagonus/I. canisuga*. In case of *D. reticulatus*, only adult stages were sent in. This is consistent with the fact that juvenile stages are nidicolous, live in close association with small mammals and have a relatively short seasonal activity period in summer with an engorgement period of 12–19 days for larvae and 3–4 days for nymphs [14].

### Infestation intensity and attachment duration

Every third cat (31.0%), but only every fifth dog (20.9%), was infested with multiple ticks. As half of the infested cats (2294/4248; 54.0%) were described as mousers, they probably spend more time in contact with vegetation and wildlife compared to dogs typically being walked by their owner for a limited amount of time. The maximum number of reported ticks from one cat was 54 specimens, all identified as *I. hexagonus* (4 females and 50 nymphs). This seems credible as contact with a hedgehog or its nest can result in such high-level infestation, especially concerning nymphs [14]. Nevertheless, the proportion of multiple infestations caused by *I. hexagonus* was never significantly larger compared to *I. ricinus*, which may have been expected because of the species’ biology, i.e. the nest adapted way of life.

Regarding multiple infestations of dogs, a Labrador-Husky mix was infested with 96 specimens (93 adult *I. ricinus* and 3 adult *D. reticulatus*). This very high infestation intensity was unexpected as, in contrast to high tick burdens caused by *R. sanguineus* in southern European countries [45], such a high infestation intensity of dogs, especially concerning only adult stages, is uncommon for the sampling region and was an exception in our dataset. It cannot entirely be ruled out that the sender added ticks from multiple animals into the same tube, but due to the extremely dense undercoat of this particular breed it is also conceivable that the ticks were missed by the owner and accumulated over time.

While cats had more multiple infestations during the periods of high tick activity, in dogs single infestations dominated the whole year round. In addition to the differences in host behaviour mentioned above, another possible reason for this pattern may be that owners are recognising tick infestations in dogs faster than in cats, resulting in removal of many ticks. This may be related to the often closer physical relationship of owners and dogs, including long and intensive grooming that often is not possible with free-roaming cats because of their more independent way of life. However, the average attachment time of 82.73 h for *I. ricinus* females on cats was only slightly higher compared to dogs (78.76 h), so the often more intimate relationship of owners to dogs did not reduce tick attachment time to an acceptable level compared to cats. These long attachment durations indicate a substantial risk of TBD transmission. Most pathogens, including *Anaplasma phagocytophilum* and *Borrelia* spp., are transmitted after 24–48 h of tick attachment [46, 47]. Regarding *B. canis*, the process of sporogony in the salivary glands takes about 48 h before transmission can occur [48]. However, an experimental approach showed that earlier transmission with pre-activated *D. reticulatus* specimens is possible within 8 h of infestation [49]. Thus, the long attachment durations observed in the current study show that visual inspection is an inadequate measure of tick control. Only for animals with minimal infection risk, meaning animals with limited or no outdoor access, may visual examination and acaricide treatment only after proven infestation be sufficient. Since no experimental data exist on the relationship between attachment duration and morphometric dimensions of *D. reticulatus*, the average engorgement time of *D. reticulatus* could not be determined in the present study. Nevertheless, a similar average engorgement duration as for *I. ricinus* can be assumed, considering the fact that almost 60% of specimens were visually assessed as partially or fully engorged.

### Seasonal abundance

Regarding seasonality, most *I. ricinus* specimens were collected in May/June of both sampled years. The lower absolute number of ticks received in May/June 2020 compared to 2021 is related to the fact that the study started in April 2020 and recruiting of participants was still ongoing until October 2020. Nevertheless, the average monthly number of ticks per actively participating veterinarian correlates with the total number of received ticks per month. The observed spring peak represents the well-known activity pattern of *I. ricinus* [50–52]. *Dermacentor reticulatus* collections showed two peaks, one in autumn 2020 and a slightly smaller one in early spring 2021. This pattern also corresponds to the literature [51–53], although some studies also report a higher spring than autumn peak [35, 54, 55].

Overall, there is a complementary activity pattern of *I. ricinus* and *D. reticulatus*, i.e. *D. reticulatus* generally shows high activity in the cooler months of the year, when activity of *I. ricinus* is rather low. Combined with the spread of *D. reticulatus*, this leads to an increased risk of tick infestation in the colder months of the year, especially for dogs. Nevertheless, winter activity of both tick species was evident. In particular, it was apparent for *D. reticulatus*, which is known to be more cold-tolerant than *I. ricinus* [20]. For *I. ricinus*, winter activity has been reported more sporadically, especially under mild circumstances [19]. According to data from the German weather service [56], the meteorological winter 2020/2021 and the preceding winters were unusually mild, with an average temperature of 1.8 °C, deviating by + 1.6 °C from the previous long-term mean (1961–1990). These mild conditions were probably conducive to a constant winter activity of both species, with a lack of, delayed or early termination of diapause [57]. In February 2021, a cold period lasting approximately 2 weeks with temperatures around − 20 °C and heavy snowfall affected large parts of Germany, which however did not seem to affect the activity of *I. ricinus* and *D. reticulatus* in the following spring, given the observed spring peak of submissions. In the face of future climate scenarios with increasingly mild winters, it is important to stress that tick winter activity no longer reflects sporadic or coincidental findings. Indeed, both questing *D. reticulatus* and *I. ricinus* were found on the vegetation during all winter months in 2021/2022 (unpublished own data). This finding in combination with the observed year-round tick infestation of dogs and cats in the study presented here shows that the traditional “standard treatment period” from April to October, covering the main activity period of *I. ricinus*, is outdated. Therefore, since both tick species transmit relevant pathogens, effective tick control in dogs and cats should no longer be limited to certain months but should be practiced all year round.

## Conclusions

Compared to previous surveys, the present large-scale study revealed marked differences in tick exposure between dogs and cats in Germany and Austria. While *I. hexagonus* remains the second most frequent tick species affecting cats, almost every fifth tick collected from dogs was identified as *D. reticulatus*. Therefore, the dramatic range expansion of *D. reticulatus* is confirmed once more, leading to an increasing risk of infection with *B. canis*. Adequate tick control, considering the shorter duration of action of several acaricides against *D. reticulatus*, is therefore indispensable and may limit the further spread of canine babesiosis, which is already on the rise in Germany. Moreover, the high percentage of multiple infestations and long average attachment duration of more than 3 days indicate a substantial risk of TBD transmission and the inadequacy of visual tick control in animals with regular outdoor access. Although the infestation risk still seems to be highest in the species-specific periods of main activity, winter activity of *D. reticulatus*, and to a lesser extent also *I. ricinus*, was observed. Because of the complementary and expanding activity patterns of these two tick species, no time of the year can be regarded as a period of negligible tick infestation risk anymore and a year-round use of licensed acaricides is therefore advocated in dogs and cats. Thorough counselling of pet owners by veterinarians regarding product choice, correct administration and duration of action is essential to protect animals successfully against health-threatening TBDs.

## Supplementary Information

**Additional file 1: Table S1. **Infestation rates with the most frequently collected tick species among tick-infested dogs and cats in the different German and Austrian federal states (number of infested hosts/% of all examined).

**Additional file 2: Table S2. **Overview of the distribution of the most frequently collected tick species from cats and dogs over the Austrian federal states (number per tick species/% of total ticks).

## Data Availability

Data supporting reported results are contained within the article. Further data can be obtained from the corresponding author on reasonable request.

## Abbreviations

TBD: Tick borne diseases
SD: Standard deviation
