# Supplementary material for: Year-round tick exposure of dogs and cats in Germany and Austria: results from a tick collection study
Source: Parasit Vectors. 2023 Feb 16;16:70. doi: 10.1186/s13071-023-05693-5 (PMC9933410; doi:10.1186/s13071-023-05693-5)
Supplement: Supplementary file 1 — Additional file 1: Table S1. Infestation rates with the most frequently collected tick species among tick-infested dogs and cats in the different German and Austrian federal states (number of infested hosts/% of all examined). [file 13071_2023_5693_MOESM1_ESM.docx]

**Additional file 1: Table S1:** Infestation rates with the most frequently collected tick species among tick-infested dogs and cats in the different German and Austrian federal states (number of infested hosts/% of all examined).

|  | **Dogs** | | | | **Cats** | | | |
| --- | --- | --- | --- | --- | --- | --- | --- | --- |
|  | **Examined dogs** | ***I. ricinus*** | ***D. reticulatus*** | ***I. hexagonus*** | **Examined cats** | ***I. ricinus*** | ***D. reticulatus*** | ***I. hexagonus*** |
| **Germany** |  |  |  |  |  |  |  |  |
| Baden-Württemberg | 593 | 530/89.38% | 27/4.55% | 9/1.52% | 392 | 348/88.78% | 1/0.26% | 33/8.42% |
| Bavaria | 644 | 600/93.17% | 26/4.04% | 18/2.80% | 710 | 644/90.70% | 1/0.14% | 44/6.20% |
| Berlin | 94 | 64/68.09% | 27/28.72% | 2/2.13% | 36 | 33/91.67% | 1/2.78% | 0/0.00% |
| Brandenburg | 438 | 203/46.35% | 318/72.60% | 5/1.14% | 269 | 222/82.53% | 29/10.78% | 9/3.35% |
| Bremen | 73 | 68/93.15% | 0/0.00% | 4/5.48% | 23 | 22/95.65% | 0/0.00% | 1/4.35% |
| Hamburg | 31 | 30/96.77% | 0/0.00% | 0/0.00% | 19 | 19/100% | 0/0.00% | 0/0.00% |
| Hesse | 426 | 346/81.22% | 85/19.95% | 6/1.41% | 202 | 192/95.05% | 2/0.99% | 5/2.48% |
| Lower Saxony | 892 | 812/91.03% | 53/5.94% | 12/1.35% | 500 | 476/95.20% | 0/0.00% | 20/4.00% |
| Mecklenburg-Western Pomerania | 144 | 131/90.97% | 11/7.64% | 2/1.39% | 101 | 95/94.06% | 0/0.00% | 5/4.95% |
| North Rhine-Westphalia | 867 | 838/96.66% | 5/0.58% | 16/1.85% | 381 | 362/95.01% | 1/0.26% | 14/3.67% |
| Rhineland-Palatinate | 379 | 349/92.08% | 24/6.33% | 4/1.06% | 246 | 233/94.72% | 2/0.81% | 9/3.66% |
| Saarland | 77 | 72/93.51% | 7/9.09% | 0/0.00% | 67 | 63/94.03% | 0/0.00% | 4/5.97% |
| Saxony | 360 | 218/60.56% | 88/24.44% | 6/1.67% | 270 | 279/87.78% | 2/0.74% | 8/2.96% |
| Saxony-Anhalt | 363 | 129/35.54% | 213/58.68% | 5/1.38% | 168 | 148/88.10% | 5/2.98% | 13/7.74% |
| Schleswig-Holstein | 300 | 294/98.00% | 0/0.00% | 2/0.67% | 210 | 207/98.57% | 0/0.00% | 2/0.95% |
| Thuringia | 102 | 294/80.39% | 21/20.59% | 6/5.88% | 129 | 100/77.52% | 0/0.00% | 20/15.50% |
| **Total** | **5,783** | **4,766/82.41%** | **905/15.65%** | **97/1.68%** | **3,723** | **3,401/91.35%** | **44/1.18%** | **187/5.02%** |
| **Austria** |  |  |  |  |  |  |  |  |
| Burgenland | 65 | 49/75.38% | 22/33.85% | 0/0.00% | 100 | 97/97.00% | 2/2.00% | 0/0.00% |
| Carinthia | 0 | 0/0.00% | 0/0.00% | 0/0.00% | 0 | 0/0.00% | 0/0.00% | 0/0.00% |
| Lower Austria | 61 | 56/91.80% | 0/0.00% | 2/3.28% | 68 | 65/95.59% | 0/0.00% | 3/4.41% |
| Upper Austria | 61 | 58/95.08% | 0/0.00% | 0/0.00% | 1 | 1/100% | 0/0.00% | 0/0.00% |
| Salzburg | 50 | 46/92.00% | 0/0.00% | 2/4.00% | 58 | 57/98.28% | 0/0.00% | 0/0.00% |
| Styria | 42 | 41/97.62% | 0/0.00% | 1/2.38% | 73 | 73/100% | 0/0.00% | 0/0.00% |
| Tyrol | 22 | 2./100% | 0/0.00% | 0/0.00% | 14 | 14/100% | 0/0.00% | 0/0.00% |
| Vorarlberg | 0 | 0/0.00% | 0/0.00% | 0/0.00% | 0 | 0/0.00% | 0/0.00% | 0/0.00% |
| Vienna | 4 | 4/100% | 1/25.00% | 0/0.00% | 1 | 1/100% | 0/0.00% | 0/0.00% |
| **Total** | **305** | **276/90.49%** | **23/7.54%** | **5/1.64%** | **315** | **308/97.78%** | **2/0.63%** | **3/0.96%** |
