# Supplementary material for: Year-round tick exposure of dogs and cats in Germany and Austria: results from a tick collection study
Source: Parasit Vectors. 2023 Feb 16;16:70. doi: 10.1186/s13071-023-05693-5 (PMC9933410; doi:10.1186/s13071-023-05693-5)
Supplement: Supplementary file 2 — Additional file 2: Table S2. Overview of the distribution of the most frequently collected tick species from cats and dogs over the Austrian federal states (number per tick species/% of total ticks). [file 13071_2023_5693_MOESM2_ESM.docx]

**Additional file 2: Table S2:** Overview of the distribution of the most frequently collected tick species from cats and dogs over the Austrian federal states (number per tick species/% of total ticks).

|  | **Dogs** | | | | **Cats** | | | |
| --- | --- | --- | --- | --- | --- | --- | --- | --- |
|  | ***I. ricinus*** | ***D. reticulatus*** | ***I. hexagonus*** | **Total** | ***I. ricinus*** | ***D. reticulatus*** | ***I. hexagonus*** | **Total** |
| Burgenland | 110/77.46% | 31/21.83% | 0/0.00% | 142/94.3% | 201/94.37% | 12/5.63% | 0/0.00% | 213/96.38% |
| Carinthia | 0/0.00% | 0/0.00% | 0/0.00% | 0/0.00% | 0/0.00% | 0/0.00% | 0/0.00% | 0/0.00% |
| Lower Austria | 73/97.33% | 1/1.33% | 2/2.67% | 75/98.68% | 108/98.18% | 0/0.00% | 3/2.73% | 110/99.10% |
| Upper Austria | 91/97.85% | 0/0.00% | 2/2.15% | 93/96.88% | 1/100% | 0/0.00% | 0/0.00% | 1/100.00 |
| Salzburg | 66/100% | 0/0.00% | 0/0.00% | 66/98.51% | 124/100% | 0/0.00% | 0/0.00% | 124/97.64% |
| Styria | 52/96.30% | 0/0.00% | 2/3.70% | 54/98.18% | 205/100% | 0/0.00% | 0/0.00% | 205/98.56% |
| Tyrol | 141/100% | 0/0.00% | 0/0.00% | 141/97.92% | 66/98.51% | 0/0.00% | 0/0.00% | 67/100% |
| Vorarlberg | 0/0.00% | 0/0.00% | 0/0.00% | 0/0.00% | 0/0.00% | 0/0.00% | 0/0.00% | 0/0.00% |
| Vienna | 4/100% | 1/25.00% | 0/0.00% | 4/80.00% | 4/100% | 0/0.00% | 0/0.00% | 4/100% |
| **Total*** | **537/93.39%** | **33/5.74%** | **6/1.04%** | **575/96.96%** | **702/96.96%** | **12/1.66%** | **3/0.41%** | **724/97.97%** |

* Percentages refer to the whole of Austria
